# Equine Standing Multidetector Computed Tomography of the Distal Thoracic Limb and Tarsus Has a Lower Cumulative Radiation Dose than Digital Radiography

**DOI:** 10.1111/vru.70049

**Published:** 2025-05-26

**Authors:** Julia L. Gaida, Tim Steinberg, Susanne M. Stieger‐Vanegas, Roswitha Merle, Christoph J. Lischer

**Affiliations:** ^1^ Tierklinik Luesche GmbH Bakum Germany; ^2^ Department of Clinical Sciences Carlson College of Veterinary Medicine Oregon State University Corvallis USA; ^3^ Clinic for Horses General Surgery and Radiology School of Veterinary Medicine Freie Universität Berlin Berlin Germany

**Keywords:** MDCT, DR, imaging, dosimetry, horse

## Abstract

Recent technological advancements in CT have improved the ability to scan standing sedated horses. However, the impact of radiation exposure on veterinary staff while scanning the extremities of standing horses using this technique, compared with digital radiography (DR), remains unknown. This study compares the radiation exposure of imaging technicians assisting with multidetector CT (MDCT) and DR of the distal thoracic limb and tarsus in standing horses. Personal dose equivalent was measured on four body locations: thyroid gland, gonads, hand, and feet. Images of the distal thoracic limb (*n* = 12) and tarsus (*n* = 12) were obtained from 24 Warmblood horses using DR and MDCT. The DR included four views of the front fetlock (dorsopalmar, lateromedial, dorso45lateral‐palmaromedial oblique, and dorso45medial‐palmarolateral oblique), three views of the front foot (dorsopalmar, lateromedial and dorso65proximal‐palmarodistal oblique) and four views of the tarsus (dorsoplantar, lateromedial, dorso45lateral‐plantaromedial oblique and dorso45medial‐planterolateral oblique). The MDCT scans included the distal metacarpus to the foot and the tarsus. Noninferiority testing showed lower radiation exposure to the imaging technician during MDCT of the distal thoracic limb and tarsus compared with DR, measured at the thyroid gland, hand, and feet. The radiation exposure to the gonads during MDCT of the thoracic limb was significantly higher than with DR. Nevertheless, the lower cumulative radiation exposure for the assisting imaging technician during MDCT compared with DR suggests that the tested MDCT setup enables advanced imaging of the distal limb in standing sedated horses, offering both reduced radiation exposure and avoiding the patient‐related risks of general anesthesia.

## 1 Introduction

Digital radiography (DR) has been used for decades and represents the first‐line imaging modality to identify orthopedic disease in horses, especially when osseous lesions are suspected. The advent of CT in equine veterinary medicine has significantly advanced diagnostic imaging. Modern CT technology offers submillimeter slices (currently as thin as 0.5 mm), providing high osseous detail [1]. CT offers several advantages over radiography, including summation‐free imaging and detailed soft tissue visualization [2]. While MRI is often regarded as the preferred modality for detecting soft tissue pathologies, recent studies indicate that CT is equally effective in identifying superficial digital flexor tendon pathology [3]. Advanced CT systems now feature dual energy capabilities, enabling the detection of clinically relevant conditions such as bone edema, which was previously only diagnosable with MRI [4]. Additionally, CT data can be used to create three‐dimensional models, facilitating surgical planning [1, 5, 6]. Overall, CT is a highly valuable diagnostic modality in equine orthopedics, enhancing visualization of abnormalities in the distal limb that were previously undetectable on radiographs, even with special radiographic views [7].

General anesthesia in horses is associated with serious neuromuscular, respiratory, systemic, or cardiovascular complications, resulting in a mortality rate of approximately 1% in healthy horses [8]. In the past decade, several adaptions in CT scanner and room configurations have enabled CT scanning of the head, neck, and distal extremities in standing sedated horses, thus optimizing patient safety by avoiding general anesthesia [7, 9, 10]. Some options were initially limited to scanning only the distal extremities, or the head and neck of the horses, and were often restricted to cone‐beam CT (CBCT) rather than fan‐beam CT [11, 12, 13]. Although CBCT units are practical and less expensive, they have limitations compared with fan‐beam multidetector CT (MDCT), including lower image quality, higher exposure settings, and increased susceptibility to motion‐related artifacts [14, 15]. Newer fan‐beam CT systems have overcome these limitations, allowing for helical CT scans in standing, sedated horses encompassing the distal limb up to the radius/tibia and the head and neck. These systems maintain the same radiation dose, speed, spatial, and soft tissue resolution as when used in other setups. In some advanced equine CT setups, this capability is achieved by mounting a large‐bore CT scanner on a movable platform. Imaging patients under sedation instead of general anesthesia is a significant advantage, as it eliminates anesthesia‐associated mortalities [16].

During standing radiographic image acquisition in horses, imaging technicians must stay close to the sedated patient to monitor and hold both the patient and the imaging equipment, resulting in radiation exposure to the veterinary staff. Safety standards for radiation protection require staff to wear protective equipment, including lead aprons, thyroid protectors, leaded protection glasses, and gloves, when handling ionizing radiation [17]. Additionally, mechanical cassette holders are used to position the X‐ray plate, thereby increasing the distance between the person holding the cassette and the primary beam. Radiation exposure to veterinary staff during routine radiographic examinations of the equine distal limb has been measured, showing moderate exposure to the torso of the staff (median personal‐equivalent dose below 2 µSv) [18]. During CBCT of the distal limb, radiation exposure at a 1.9 m distance from the center of the gantry was measured at 0.5 µSv with lead protective equipment and approximately 12 µSv without any lead protection [10]. MDCT of the distal limb revealed radiation doses of 5.36 µGy (equivalent to 5.36 µSv) for imaging the thoracic limb and 0.15 µGy (equivalent to 0.15 µSv) for the pelvic limb, measured below a lead apron at distances of 1.17 and 2.96 m, respectively [19]. However, radiation exposure during standing MDCT exams of the distal limb of standing horses, compared with DR, is currently unknown. To reduce radiation exposure to personnel during CT scans of standing horses, additional safety measures are being explored with newer CT systems. These measures include the use of lead curtains in front of the CT system, lead shields in front of the personnel, and pulley systems to hold the horse's legs in place [13, 15, 20].

The use of ionizing radiation, such as in CT imaging, poses potential health risks due to its deterministic (threshold dose exposure) and stochastic (long‐term, low‐level exposure) effects, which can significantly impact the human body, including the risk of cancer [21]. Health‐related consequences from long‐term low‐dose radiation, such as those encountered in occupational exposure situations, are often not immediately noticeable [22, 23]. However, research has demonstrated a linear increase in mortality and cancer incidence with increasing low‐dose radiation exposure [24, 25]. Therefore, national (e.g., Germany) and international (e.g., International Commission on Radiological Protection) radiation regulations mandate constant monitoring of occupational exposure situations and provide dose limits (Table 1) for the whole body (effective dose) and specific body locations (personal dose equivalent). The effective dose is used to estimate the risk of stochastic effects, while the dose equivalent monitors radiation exposure above a certain dose threshold, indicating the risk for deterministic effects [20]. According to these regulations, the effective dose limit for the whole body is set at 20 mSv per year, and the equivalent dose limit for hands, feet, and skin is set at 500 mSv per year [26, 27]. Due to the increased prevalence of cataracts associated with occupational radiation exposure, an equivalent dose limit of 150 mSv per year is recommended for the lenses [26, 27, 28, 29].

**TABLE 1:** Recommended dose limits in planned exposure situations of the International Commission on Radiological Protection (ICRP).

Type of limit	Occupational
Effective dose	20 mSv per year, averaged over defined periods of 5 years
Annual equivalent dose in	
Lens of the eye	150 mSv
Skin	500 mSv
Hands and feet	500 mSv

The personal dose equivalent (H_p_) is directly measurable by placing a dosimeter over the organ of interest to assess the radiation dose at a specific tissue depth (d). The personal dose equivalent for the gonads and the thyroid gland is assessed at a tissue depth of 10 mm [H_p_ (10)], for the lenses at a tissue depth of 3 mm [H_p_ (3)], and for the skin, hands and feet at a tissue depth of 0.07 mm [H_p_ (0.07)]. The risk of tissue reactions depends on the effective dose, which is a calculated value and not directly measurable. The effective dose for the whole body is equal to the personal dose equivalent for a depth of 10 mm for the body part receiving the highest exposure [26].

Currently, the exact radiation dose received by veterinary staff during MDCT scans of the distal limb in standing, sedated horses using newer technical setups with a lead shield at the CT gantry opening is not known. The study aims to measure the radiation exposure experienced by imaging technicians when using a helical MDCT system for scanning standing horses and to compare these values to those obtained during digital radiography (DR) of the same anatomical area. Additionally, this study seeks to determine the maximum number of standing MDCT examinations a veterinary imaging technician can assist in before reaching the occupational exposure dose limits.

We hypothesize that radiation exposure to veterinary imaging technicians during MDCT scans of the distal thoracic limb and the tarsus will not exceed the exposure levels during equivalent radiographic examinations of the same body area using DR.

## 2 Material and Methods

### 2.1 Selection and Description of Patients

The study was prospective, observational, and descriptive. Warmblood horses admitted to a private equine clinic in Germany for lameness examinations or prepurchase exams from May 2021 to June 2022 were enrolled. Consent from the owner was not necessary, as the study design did not alter the quality or quantity of diagnostic procedures performed on the patients, thus positing no harm to the horses. The signalment and history of each horse were recorded. Inclusion criteria required that the horse underwent either DR or standing MDCT examination of the distal thoracic extremity and tarsus, encompassing the entire area. For the distal thoracic extremities, imaging had to extend from the distal metacarpus to the toe and for the tarsus from the distal tibia to the proximal metatarsus. A study was considered complete if the entire distal thoracic extremity and the entire tarsus were scanned.

### 2.2 Dosimetry of Personnel Involved in MDCT or DR Procedure

**FIGURE 1:**
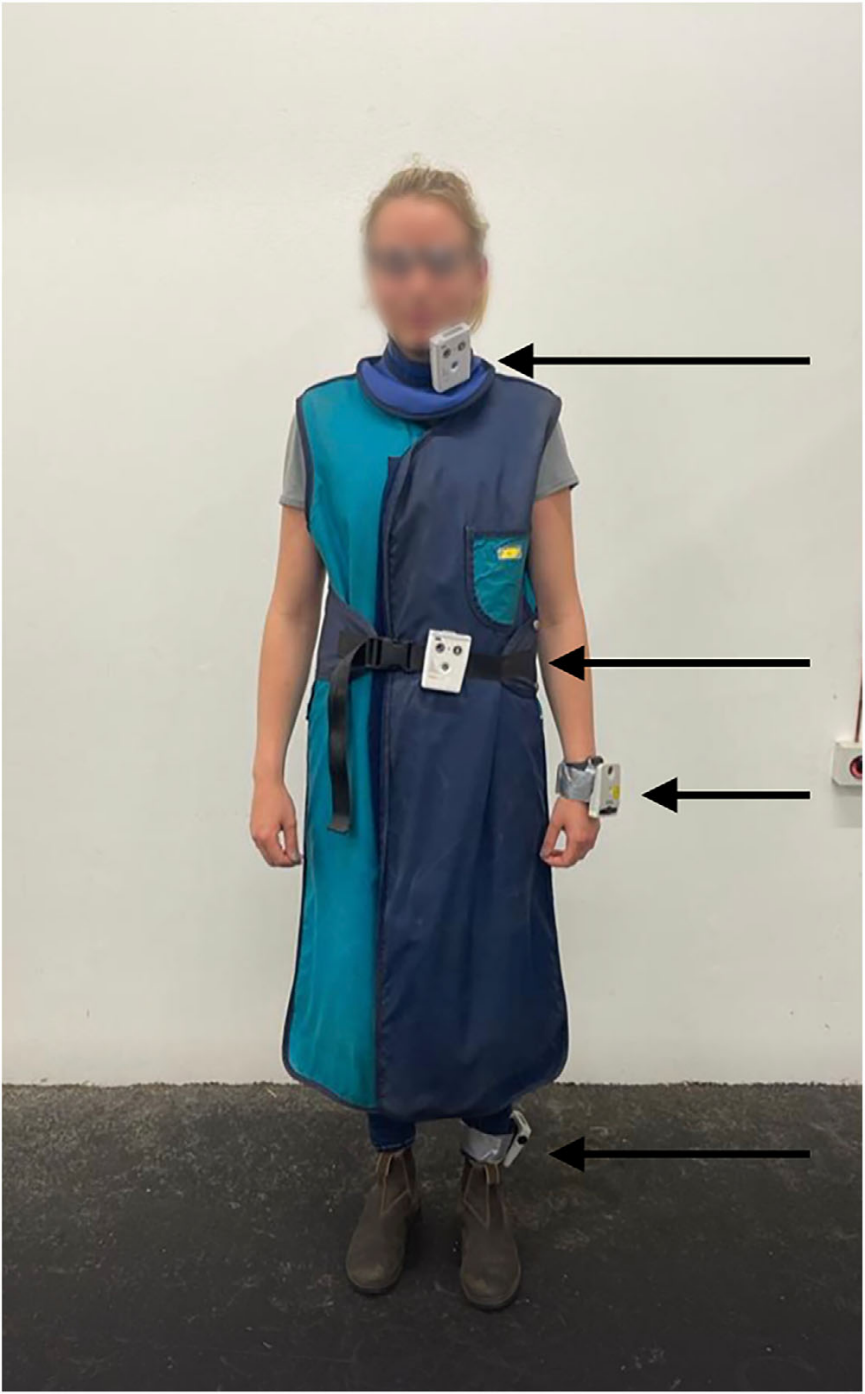
Dosimeter locations during the acquisition of MDCT scans of the distal thoracic limb and the tarsus. Arrows indicate dosimeter locations (L_thyroid, L_gonads, L_hands, L_feet). Radiation protection gear includes leaded eyewear, a thyroid protector, and a lead apron. Note: The locations for L_hands and L_feet varied between the left and right sides depending on the radiographed side of the Horse.

**FIGURE 2:**
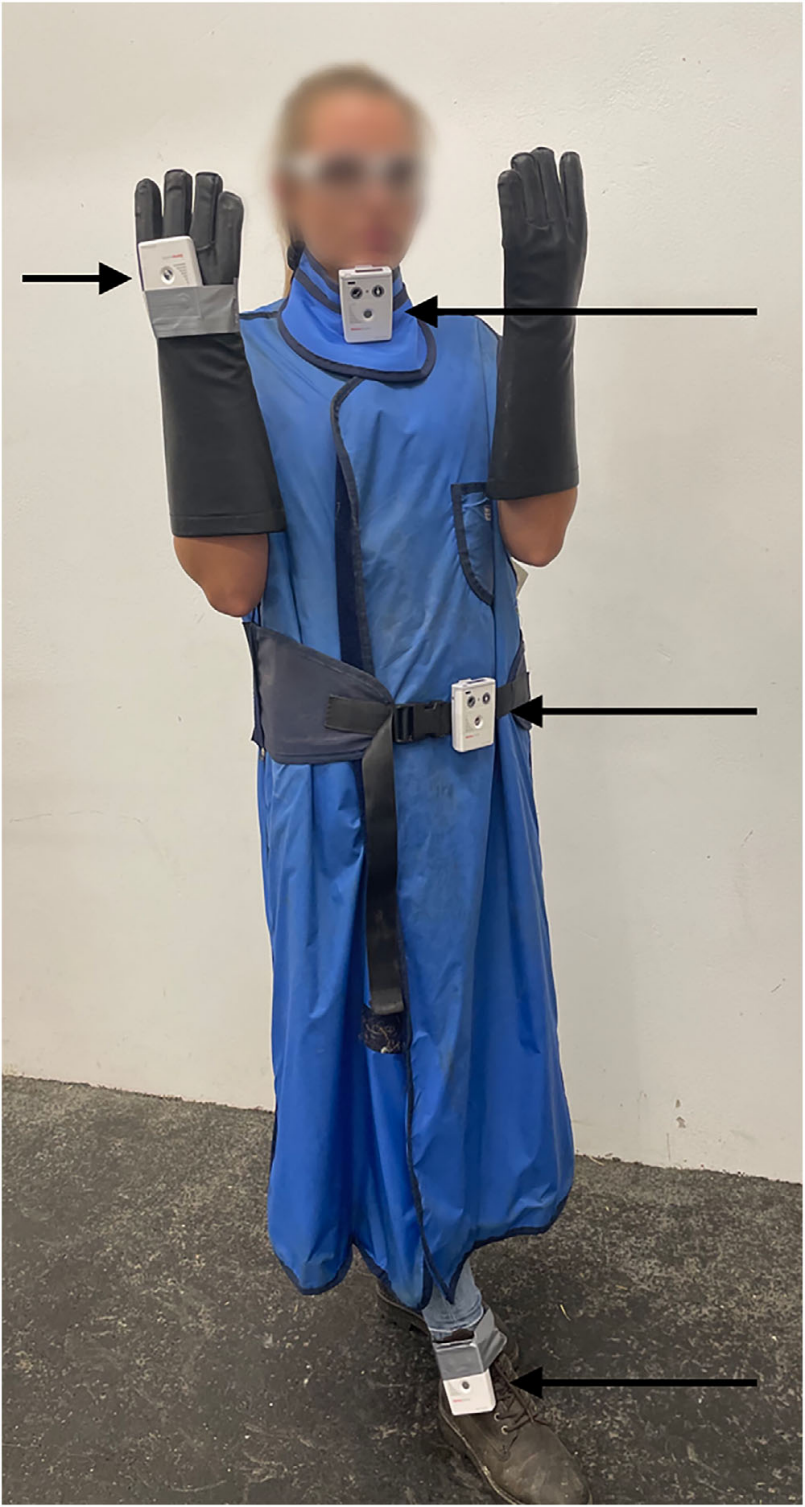
Dosimeter locations during DR imaging of the distal thoracic extremity and tarsus. Arrows indicate dosimeter locations (L_thyroid, L_gonads, L_hands, L_feet). Radiation protection gear includes leaded eyewear, a thyroid protector, lead gloves, and a lead apron. Note: The locations L_hands and L_feet varied between the left and right sides depending on the radiographed side of the horse.

**FIGURE 3:**
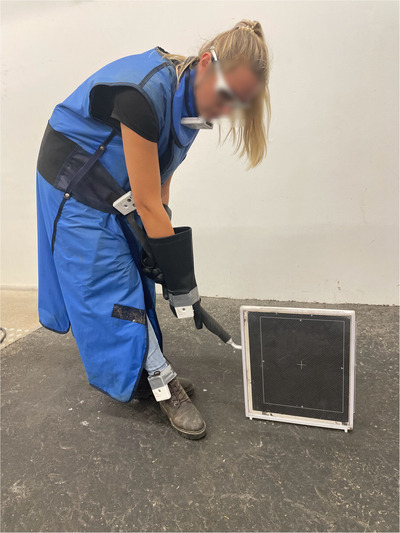
Plate holder equipped with radiation protection gear and personal dosimeters during DR imaging of the distal thoracic extremity and tarsus.

The imaging technicians assisting with the MDCT and DR procedures were equipped with a lead apron (lead equivalent: 0.5 mm), a thyroid protector (lead equivalent: 0.5 mm), and leaded eyewear (Figure 1) to ensure compliance with radiation protection guidelines [17]. Lead gloves were not used for the imaging technician during MDCT because they would restrict control over the patient and the rope. According to radiation safety guidelines for radiographic examinations in Germany, lead gloves are required only when there is a risk of the hands being close to the primary beam, which was not applicable in this scenario [17]. However, for the DR examinations, the technician holding the flat panel used lead gloves (Figure 2) and an X‐ray cassette holder on a distance stick (Figure 3).

The study design for dosimetry was based on previous research [18, 30, 31]. Four electronic personal dosimeters (Thermo Scientific EPD TruDose, Erlangen, Bayern, Germany) were positioned at specific locations on the imaging technician's body (Figure 1). One dosimeter was placed on top of the thyroid protector near the imaging technician's thyroid gland (Location_thyroid, L_thyroid). The second dosimeter was positioned near the gonads on top of the lead apron (Location gonads, L_gonads). The third dosimeter was placed dorsally on the operating hand holding the rope of the quick‐release pulley system (Location hand, L_hand). This dosimeter was located on the left hand when scanning the right thoracic‐ and pelvic limbs and on the right hand when scanning the contralateral limbs. The fourth dosimeter was positioned on the cranial aspect of the tibia, which was not shielded by the lead apron (Location feet, L_feet). This dosimeter was located on the tibia closest to the gantry.

To determine effective doses for the location of the thyroid gland (L_thyroid) and the gonads (L_gonads) in this study, the personal dose equivalent for a depth of 10 mm [H_p_ (10)] was assessed. For the assessment of the equivalent dose to the skin of the hands (L_hands) and leg (L_feet), the personal dose equivalent at a depth of 0.07 mm [H_p_ (0,07)] was evaluated [26]. The highest measured personal dose equivalent H_p_ (10) between L_thyroid and L_gonads was considered representative of the effective dose for the whole body and was compared with the exposure dose limit of 20 mSv per year [32]. The personal dose equivalent values H_p_ (0.07) measured at L_hands and L_feet were compared with the equivalent dose limits of 500 mSv per year. The provided occupational exposure dose limits for the effective dose and for the equivalent doses H_p_ for specific body locations (Table 3) were used to calculate the maximum number of annual scans [26, 27]. The cumulative dose was calculated by summing the average levels obtained from the four measured locations during imaging of the distal thoracic limb and the tarsus (i.e., MDCT distal thoracic extremity and MDCT tarsus; DR distal thoracic extremity and DR tarsus) combined.

### 2.3 Data Analysis and Recording

Each dosimetry measurement was conducted on an individual MDCT scan or a single acquisition for each radiographic study obtained. In cases where patient movement necessitated any additional MDCT or DR, exposure was not factored into the measurement to allow a direct comparison of the dosimetry results. The duration of each imaging examination, from the initial positioning of the horse to the completion of the MDCT scan or DR study, was timed. This timing encompassed any repeat MDCT scans or DR images as required. All dosimetry data of each imaging study were collected and electronically archived for analysis.

### 2.4 Statistical Analysis

All data analysis was conducted by a Diplomate of the European College of Veterinary Public Health (R. M.), using Microsoft Excel Version 16.66.1. Noninferiority testing was applied to compare the mean personal dose equivalent for MDCT distal thoracic extremity/tarsus with DR distal thoracic extremity/tarsus at each dosimeter location. To assess radiation exposure differences between the MDCT and DR groups, a predetermined noninferiority margin of 0.4 µSv was established based on normally distributed samples obtained during pilot dosimetry measurements on the same CT setup prior to the main study. Six dosimetry pilot measurements were performed for both MDCT and DR during routine equine diagnostic procedures (data can be requested as a supplementary appendix), with radiation exposure ranging from a minimum of 0.6 µSv to a maximum of 2.3 µSv. A 0.4 µSv difference in radiation exposure between MDCT and DR was found to be clinically relevant in terms of its potential impact on the severity of the stochastic effect. A significant and statistically relevant noninferior result was determined if the 95% confidence interval did not encompass the 0.4 µSv margin. This would suggest that radiation exposure in the MDCT distal thoracic extremity/tarsus group was either lower or at least not higher than in the DR group. Sample size calculation revealed that nine animals per group were adequate to detect an estimated difference of −0.1, assuming a standard deviation of 0.4 with a power of 81% (NCSS PASS Version 14). However, to ensure a minimum power of 80% under varied conditions, 12 animals per group were chosen for the study.

## 3 Results

### 3.1 Horses

Twelve Warmblood horses presenting with thoracic‐ and pelvic limb lameness (*n* = 12) underwent CT scans (group MDCT) of the distal thoracic limb (MDCT distal thoracic extremity; *n* = 4 left and *n* = 8 right distal thoracic limb) and tarsus (MDCT tarsus; *n* = 5 left and *n* = 7 right tarsus). The horses’ weights ranged from 500 to 700 kg, with a mean weight of 600.0 (±74.7 kg), and their mean age was 9.4 years (±3.9). Twelve Warmblood horses (*n* = 12) underwent radiographic examinations (group DR) of the distal thoracic limb (DR distal thoracic extremity; *n* = 6 left and *n* = 6 right distal thoracic limb) and the tarsus (DR tarsus; *n* = 6 left and *n* = 6 right tarsus). Their weights ranged from 500 to 700 kg (mean weight: 612.9 ± 71.1 kg), and their mean age was 8.7 ± 4.6 years.

All 24 horses were intravenously sedated for the imaging examination using 0.01–0.02 mg/kg Detomidine (Domidine, Aulendorf, Baden‐Württemberg, Germany) and 0.02–0.05 mg/kg Butorphanol (TorbugesicVet, Berlin, Germany). Sedation was administered to achieve the desired effect, aiming to minimize patient movement during image acquisition.

### 3.2 MDCT Imaging

Computed tomography was performed following established protocols [33]. MDCT scans were acquired using a 16‐row MDCT scanner (Canon Medical Aquilion Large Bore CT, gantry diameter of 90 cm, Canon Medical System GmbH Neuss, Nordrhein‐Westfalen, Germany) without the intravenous administration of iodinated contrast agent. The MDCT scanner was mounted on a moveable platform (Qalibra CT, Qalibra US Inc/Vet‐DICon GmbH, Zossen, Germany, www.qalibra.com) employing an innovative approach where the CT gantry, rather than the patient couch, is moved and positioned over the patient's limb for imaging. This technique optimizes imaging of standing patients and facilitates precise positioning. MDCT imaging was performed on the distal thoracic limb (MDCT distal thoracic extremity) from the distal metacarpus to the foot and the tarsus (MDCT tarsus) from the distal tibia to the proximal metatarsus. The following imaging parameters were utilized: 0.5 mm reconstruction interval, 512 × 512 matrix size, 70 cm field‐of‐view, 300 mm scanning distance, the helical pitch of 0.8, the gantry rotation speed of 0.5 s, 370 mA and 120 kVp, and 0.5 mm isotropic voxel scanning. Image reconstruction was performed with 0.5 mm slice thickness in transverse, sagittal, and dorsal planes using both soft tissue (FC 08) and bone (FC 30) algorithms. The scanning duration per scan was 8 s, ensuring efficient data acquisition while maintaining image quality.

**FIGURE 4:**
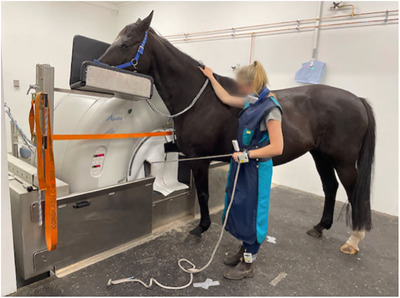
Positioning of the veterinary imaging technician and horse for MDCT imaging of the distal thoracic extremity.

Furthermore, the MDCT setup included a quick‐release pulley system, which allowed imaging technicians to position the patient's limb from the patient's side using a rope (Figure 4). The system enabled the operator to release the rope immediately if needed, ensuring the safety of the staff and the patient while preventing potential damage to the equipment. Additionally, the MDCT scanner was equipped with a custom‐designed lead curtain, with a mean lead equivalent of 1.5 mm, located in front of the CT bore to provide further radiation shielding for the personnel (Figure 4).

**FIGURE 5:**
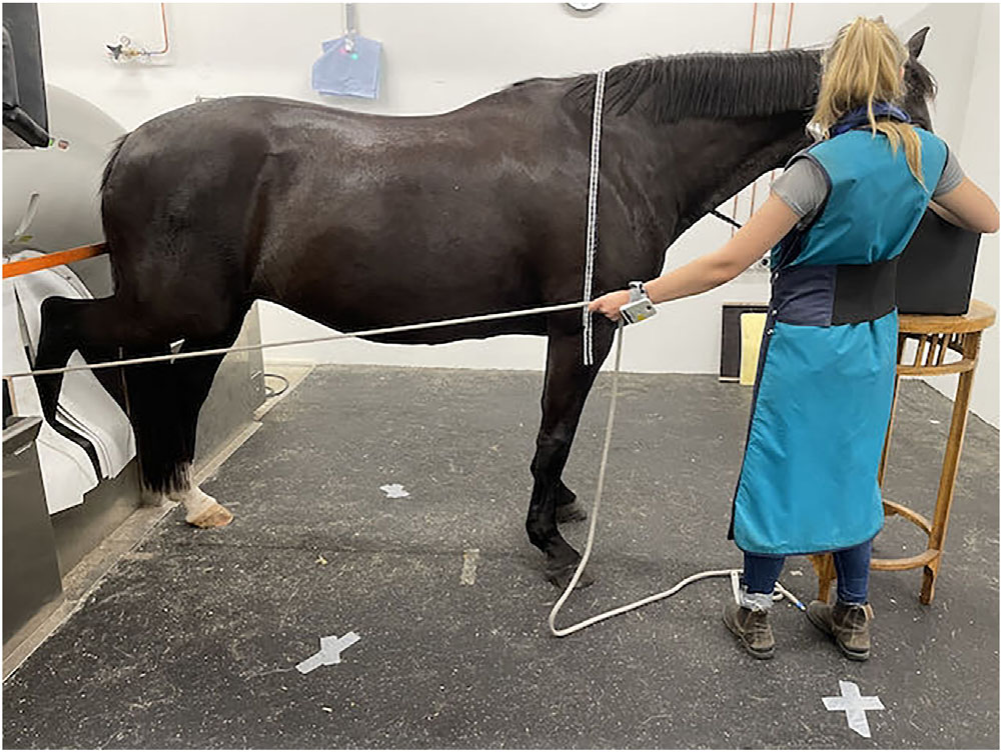
Positioning of the veterinary imaging technician and the horse for MDCT imaging of the tarsus.

During the MDCT acquisition, only one imaging technician was present inside the CT room. For scanning the distal thoracic limb, the technician positioned the distal aspect of the limb into the CT gantry while supporting the horse's head on a custom headstand. The technician held the patient's limb in position using the quick‐release pulley system (Figure 4). For scanning the tarsus, the pelvic limb was placed into the CT gantry and similarly positioned using the pulley system (Figure 5), with the horse's head again supported on a stand. The quick‐release pulley system was operated with the right hand when scanning the left thoracic and pelvic limbs and with the left hand when scanning the contralateral limbs.

**FIGURE 6:**
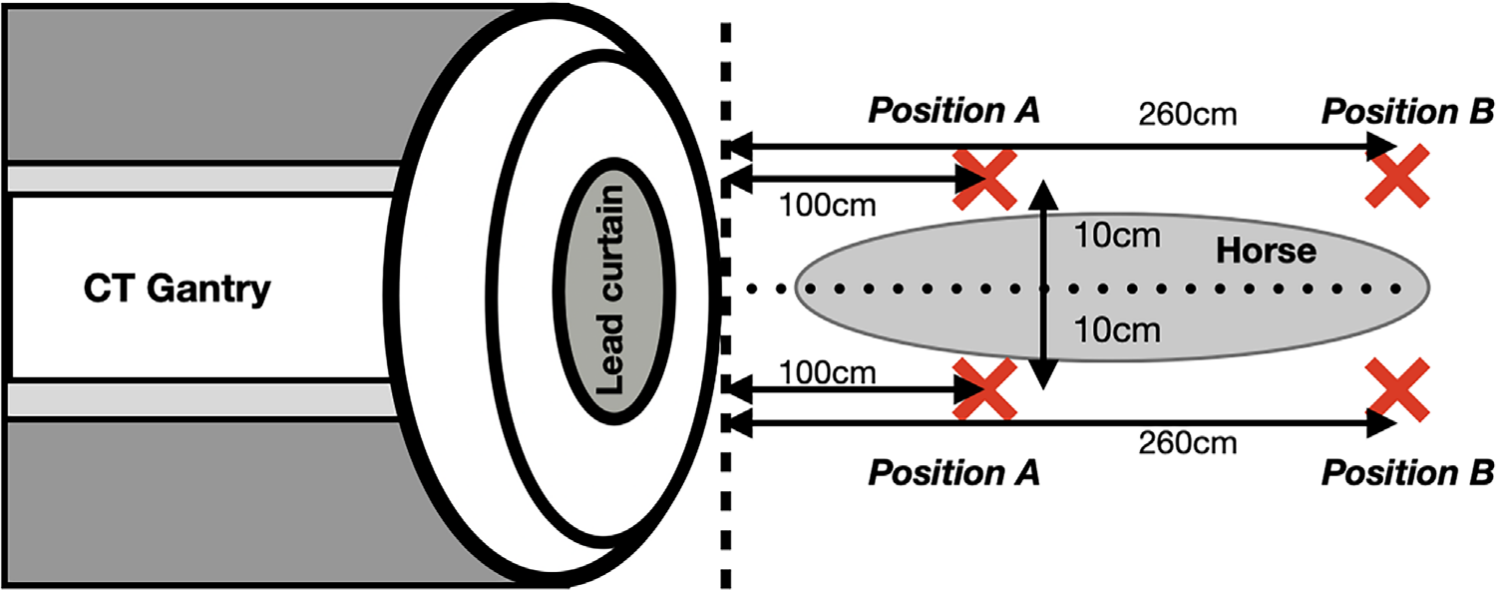
Positioning of the imaging technician during the acquisition of MDCT scans of the distal thoracic extremity (position A) and tarsus (position B). The oval demonstrates the horse's position on the midline of the CT gantry. Black arrows indicate the distance between the CT gantry and position A (100 cm, 10 cm from the midline of the CT gantry) or position B (260 cm, 10 cm from the midline of the CT gantry). The cross indicates the standing position of the imaging technician (position A or B).

Dosimetry measurements were performed with the imaging technician standing in position A for the MDCT scan of the distal thoracic extremity and in position B for the MDCT scan of the tarsus (Figure 6). Position A, located approximately 100 cm from the center of the MDCT gantry, was at the horse's left shoulder for scanning the right distal thoracic extremity and at the horse's right shoulder for scanning the left distal thoracic extremity (Figure 4). Position B, approximately 260 cm from the center of the gantry, was on the right side of the horse's head for scanning the right tarsus and on the left side for scanning the left tarsus (Figure 5). Floor markings indicated the exact standing positions of the imaging technician.

### 3.3 DR Imaging

All DR images were acquired using a commercially available X‐ray system (Gierth RHF 200 ML, Gierth X‐ray International GmbH, Riesa, Sachsen, Germany) and X‐ray plate (Rayence XMARU 1012 WCA, Rayence USA, Closter, NJ, USA; 39.5 cm × 33.7 cm × 1.8 cm). The images were obtained with a constant focus‐film distance of 60–70 cm between the generator and the X‐ray cassette. For each horse, seven radiographs were acquired of the distal thoracic limb (DR distal thoracic extremity) and four for the tarsus (DR tarsus) in accordance with the standard clinical examination protocols for the distal extremities [34]. The DR images of the distal thoracic extremity included four views of the front fetlock (dorsopalmar, lateromedial, dorso45lateral‐palmaromedial oblique, and dorso45medial‐palmarolateral oblique) and three views of the foot (dorsopalmar, lateromedial and dorso65proximal‐palmarodistal oblique). The DR tarsus images comprised four views (dorsoplantar, lateromedial, dorso45lateral‐plantaromedial oblique and dorso45medial‐planterolateral oblique). The generator settings used for these images are detailed in Table 2.

**TABLE 2:** Settings for digital radiography acquisition for distal thoracic extremity and tarsus.

Anatomic location	Radiographic view	kVp	mAs	Collimation (cm × cm)
Foot	Dorsopalmar	68	0.08	25 × 30
	Lateromedial view	68	0.08	25 × 30
	Dorso65proximal‐palmarodistal Oblique	70–72	0.12	22 × 25
Fetlock	Dorsopalmar	68	0.08	25 × 30
	Lateromedial	68	0.08	25 × 30
	Dorso45medial‐palmarolateral oblique	68	0.08	25 × 30
	Dorso45lateral‐palmaromedial oblique	68	0.08	25 × 30
Tarsus	Dorsoplantar	70–72	0.1–0.12	25 × 32
	Lateromedial	70	0.1	25 × 32
	Dorso45lateral‐planteromedial oblique	70	0.1	25 × 32
	Dorso45medial‐planterolateral oblique	70	0.1	25 × 32

Three imaging technicians were present during DR procedures: one person holding the equine patient, one holding the X‐ray plate in the cassette holder, and one operating the generator. Imaging technicians wore the same protective outfits as previously described (Figure 2). As with the MDCT acquisition, floor markings indicated the precise standing position for the technician holding the plate, who was positioned perpendicular to the X‐ray generator.

All imaging technicians were equipped with dosimeters. However, for this study, only the exposure values from the imaging technician holding the X‐ray plate were analyzed, as previous literature indicated the highest radiation exposure at this position [18]. Dosimetry was conducted similarly to the CT scans with dosimeters placed at the thyroid (L_thyroid), gonads (L_gonads), hands (L_hands), and feet (L_feet). The dosimeter on the hand (L_hands) closest to the flat panel was placed dorsally on the lead glove holding the distance stick of the cassette holder (approximate distance between hand and cassette: 20 cm, Figure 3). The dosimeter on the feet (L_feet) was placed on the cranial aspect of the left tibia of the imaging technician. For DR of the right thoracic and pelvic limbs, the dosimeter was placed on the left hand, and for the contralateral limb, it was placed on the right hand.

### 3.4 Radiation Exposure Comparison of the MDCT to DR Distal Thoracic Extremity Dosimetry Data

**FIGURE 7:**
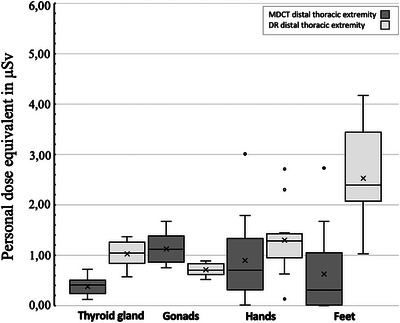
Comparison of radiation exposure during MDCT and DR imaging of the distal thoracic extremity, with four dosimeter locations (L_thyroid, L_gonads, L_hands, L_feet) on the x‐axis and personal dose equivalent in µSv on the y‐axis.

Mean radiation values comparing MDCT scans of the thoracic limb to DR are presented in Table 3 and Figure 7. Most comparisons (thyroid gland, hand, feet) indicated that MDCT is noninferior to DR. For instance, the mean values measured for L_thyroid showed a difference of −0.64 (95% confidence interval −0.74 to −0.55), indicating that in this setup, MCDT exposes the thyroid gland to lower radiation levels than DR. However, the measurements for L_gonads during MDCT and DR revealed a difference of 0.41 (95% confidence interval 0.23–0.59), suggesting that MDCT is not noninferior to DR in this setup. This means that radiation exposure to the gonads of the imaging technician is higher during MDCT of the distal thoracic extremity compared with DR.

**TABLE 3:** Personal dose equivalent in µSv at four locations including thyroid (L_thyroid), gonads (L_gonads), hand (L_hand), and feet (L_feet) during multidetector computed tomography and digital radiography of the distal thoracic extremity.

Procedure	L_thyroid		L_gonads		L_hands		L_feet	
	MDCT	DR	MDCT	DR	MDCT	DR	MDCT	DR
1	0.23	1.09	1.14	0.84	0.76	1.25	2.73	2.42
2	0.43	0.92	0.92	0.82	0.01	1.31	0.01	4.17
3	0.30	0.99	1.67	0.67	0.58	1.13	0.59	2.06
4	0.52	1.28	1.19	0.60	3.01	1.37	0.18	1.03
5	0.72	1.29	1.51	0.73	0.36	2.30	0.67	2.47
6	0.52	1.37	0.94	0.68	0.08	1.44	1.67	1.03
7	0.40	1.14	1.09	0.66	1.79	1.03	1.17	2.37
8	0.44	0.99	0.75	0.74	1.42	0.63	0.43	2.63
9	0.27	0.81	0.76	0.54	0.73	1.38	0.05	2.27
10	0.46	1.19	1.43	0.87	0.30	2.71	0.00	3.71
11	0.12	0.57	1.23	0.89	1.07	0.13	0.00	4.05
12	0.15	0.64	0.84	0.52	0.67	0.92	0.01	2.10
Mean	0.38	1.02	1.12	0.71	0.90	1.30	0.63	2.53
SD	0.17	0.25	0.30	0.12	0.85	0.68	0.85	1.02
Difference MDCT/DR mean	−0.64^a^		0.41		−0.40^a^		−1.9^a^	
95% Confidence interval	−0.74 to −0.55		0.23 to 0.59		−1.19 to 0.39		−2.88 to −0.92	

Abbreviations: MDCT, multidetector row computed tomography; DR, digital radiography; SD, standard deviation.

^a^ Statistically significant difference.

### 3.5 Radiation Exposure Comparison MDCT to DR Tarsus

**FIGURE 8:**
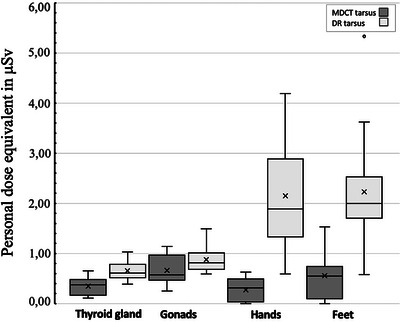
Comparison of radiation exposure during MDCT and DR imaging of the tarsus, with dosimeter location (L_thyroid, L_gonads, L_hands, L_feet) on the x‐axis and personal dose equivalent in µSv on the y‐axis.

Mean radiation values comparing MDCT scans of the tarsus to DR are presented in Table 4 and Figure 8. All comparisons demonstrated noninferiority of MDCT compared with DR for the tarsus, indicating that radiation exposure to all four locations was lower during MDCT than during DR imaging.

**TABLE 4:** Personal dose equivalent in µSv at four locations including the thyroid (L_thyroid), gonads (L_gonads), hand (L_hand), and feet (L_feet) during multidetector computed tomography and digital radiography of the tarsus.

Procedure	L_thyroid		L_gonads		L_hands		L_feet	
	MDCT	DR	MDCT	DR	MDCT	DR	MDCT	DR
1	0.11	0.60	1.14	0.68	0.33	2.59	0.00	0.78
2	0.11	0.71	0.25	0.69	0.38	1.88	0.74	2.22
3	0.40	0.43	0.36	0.76	0.03	1.28	1.22	3.62
4	0.50	0.50	0.46	0.71	0.13	0.88	0.34	5.33
5	0.21	1.03	0.50	1.21	0.53	0.59	0.74	1.92
6	0.41	0.96	0.77	1.49	0.53	3.63	0.49	1.97
7	0.65	0.59	1.03	0.59	0.04	1.60	0.01	0.58
8	0.35	0.39	0.57	0.96	0.63	1.90	0.61	2.03
9	0.40	0.62	0.73	0.66	0.00	1.49	0.70	1.63
10	0.53	0.81	1.11	0.87	0.30	2.91	0.03	1.96
11	0.15	0.64	0.57	0.90	0.36	4.19	1.53	2.63
12	0.31	0.57	0.49	1.03	0.01	2.82	0.31	2.11
Mean	0.34	0.65	0.67	0.88	0.27	2.15	0.56	2.23
SD	0.17	0.20	0.29	0.26	0.23	1.10	0.47	1.25
Difference mean MDCT/DR	−0.31^a^		−0.21^a^		−1.87^a^		−1.67^a^	
95% Confidence interval	−0.49 to −0.13		−0.48 to 0.05		−2.55 to −1.19		−2.41 to −0.93	

Abbreviations: MDCT, multidetector row computed tomography; DR, digital radiography; SD, standard deviation.

^a^ Statistically significant difference.

The measured doses per scan for all locations during MDCT and DR of the distal thoracic extremity and the tarsus showed negligible values (Table 5). Similarly, the maximal number of scans per year until reaching the annual dose limit for the effective dose of the whole body (represented by personal dose equivalent H_p_ (10); highest H_p_ (10) measured at the location of the thyroid gland or gonads) and the dose equivalent of hands and feet ranged from 17,857 to 1,851,851. The calculated average cumulative dose was 0.61 µSv for MDCT and 1.43 µSv for DR.

**TABLE 5:** Highest measured mean personal dose equivalent H_p_ (10) and mean equivalent doses for hands and feet in µSv, with maximal annual scans until reaching the determined International Commission on Radiological Protection (ICRP) occupational exposure limits.

	Highest mean H_p_ (10) L_thyroid or L_gonads		H_p_ (0.07) L_hands		H_p_ (0.07) L_feet	
	Mean effective dose/scan	Max. annual scans	Mean equivalent dose/scan	Max. annual scans	Mean equivalent dose/scan	Max. annual scans
MDCT distal thoracic extremity	1.12	17,857	0.90	555,555	0.63	793,650
DR distal thoracic extremity	1.02	19,607	1.30	384,615	2.53	197,628
MDCT tarsus	0.67	29,850	0.27	1,851,851	0.56	892,857
DR tarsus	0.88	22,727	2.15	232,558	2.23	224,215

*Note*: **Highest measured mean personal dose equivalent H_p_ (10)** in µSv between the location of the thyroid gland and gonads (L_thyroid, L_goands) per scanning session (MDCT/DR distal thoracic extremity and tarsus), representing the mean effective dose for the whole body. **Mean personal equivalent doses** in µSv for the locations of the hands and the feet (L_hand and L_feet) per scanning session (MDCT/DR distal thoracic extremity and tarsus). **Calculated maximal annual scans** until reaching the dose limits for a single operator, comparing it to the determined ICRP occupational exposure limits (annual effective dose for the whole body: 20 mSv; annual equivalent dose for hands and feet: 500 mSv).

Abbreviations: MDCT, multidetector row computed tomography; DR, digital radiography.

### 3.6 Time and Repetitions

The mean duration for MDCT of the distal thoracic extremity, including an average of 2 scan repetitions (range: 1–3 repetitions, mean: 2.00 ± 0.74 repetitions), was 20.57 ± 1.59 min. The mean duration for DR of the distal thoracic extremity was 2.89 ± 0.89 min, including an average of 1 image retake (range: 0–3 retakes, mean: 0.92 ± 1.0) per radiographic set.

The mean duration for MDCT of the tarsus, including an average of 2 repetitions (range: 1–4 repetitions, mean: 2.33 ± 0.89), was 21.21 ± 1.11 min, and for DR imaging of the tarsus 2.10 ± 0.48 min, including an average of 1 retake (range: 0–2 retakes, mean: 1.00 ± 0.74) per radiographic set.

## 4 Discussion

This study compares radiation exposure of veterinary staff between MDCT and DR when acquiring images of the thoracic distal limb and tarsus in standing horses. The results show lower personal dose equivalent values for most locations when comparing exposure values during MDCT and DR imaging. This finding supports our hypothesis, that radiation to veterinary staff during standing MDCT scans would be at least equal to, if not lower than, DR imaging of the same body area. These results contrast with the common belief that MDCT procedures are associated with higher radiation exposure and that the MDCT scatter radiation significantly contributes to increased radiation exposure during the scan [35].

Thus, this study provides new insights, as a direct comparison of radiation exposure from MDCT procedures on standing horses to DR has not been done before. However, several studies have measured radiation exposure doses for different CT setups on standing horses. Previously reported exposure values for veterinary technicians assisting head CT on standing sedated horses show lower exposure levels at the handler's hand (1.5 µSv over 5 examinations) but higher exposure values at the thyroid cover (5.0 µSv over 5 examinations) compared with the present study [13]. In that setup, the technician handling the horse is positioned at the side of the CT machine, unlike in the present study, where they are positioned in front of the CT gantry. A more recent study investigating radiation exposure to staff when assisting standing CT of the distal limb and carpus/tarsus with a CBCT unit showed markedly higher exposure values during one image acquisition: 28–51 µSv at 0.6 m and 11.8 µSv at 1.9 m from the isocentre of the CBCT scanner, without any lead shielding of the personnel [10]. This has also been reported by a previous study, highlighting the disadvantage of high exposure settings and scatter radiation when using CBCT [14]. Similarly, an experimental study assessing radiation exposure to the lead rope handler during MDCT of the distal limb revealed substantially higher radiation doses without lead protection compared with our study, measuring 67.8 µSv at a distance of 1.17 m and 1.9 µSv at a distance of 2.96 m from the isocenter [19].

Several factors contributed to the lower exposure values to staff in our study. To enhance radiation protection, our MDCT setup included a quick‐releasing pulley system, allowing staff to operate the horse from a greater distance to the CT scanner isocenter. This follows the “As Low As Reasonable Achievable” (ALARA) principles, which state that increasing the distance from the radiation source reduces radiation exposure by the square of the distance [10, 36]. Consequently, several other studies have reported a significant reduction in radiation doses with increasing distance from the center of the gantry [10, 37]. Additionally, the integrated lead curtain in the CT setup likely reduced the scattered radiation, thereby lowering the radiation exposure to the imaging technician. However, this is an assumption, as we did not measure radiation exposure without the lead curtain. A similar attempt to reduce radiation exposure was made in fluoroscopy‐assisted cardiac resynchronization therapy in human medicine using a lead‐shielding drape [20]. In that study, scatter radiation measured at the height of the gonads was reduced by 74% to 84%, depending on the pulse rate and the positioning relative to the lead‐shielding drape. However, scatter radiation measured slightly off from the lead shielding drape, as would be the case for an assistant physician during the procedure, was only decreased by 14% to 19%. While the radiation settings and isodose curves in fluoroscopy differ from the current study, the results of using lead shielding to decrease the fluoroscopy‐derived scatter radiation highlight the potential for improving radiation safety. Implementing more lead shielding in MDCT procedures could similarly enhance radiation protection for veterinary staff.

One exception to the general trend of lower radiation exposure with MDCT compared with DR was the associated higher exposure for the gonads during MDCT of the distal thoracic limb. The highest MDCT dose values were measured at the gonad's location during the MDCT scan of the distal thoracic limb (1.12 µSv). Several factors explain this difference, including the shorter distance of the imaging technician to the gantry and isocenter of the CT scanner and incomplete shielding from the lead curtain of the CT scanner at the level of the gonads. The latter is particularly challenging to avoid because the horse's limb needs to be inside the gantry, creating an opening in the lead curtain. In comparison, the center of the beam and the source of scattered radiation for DR of the thoracic distal limb is located at the level of the imaging technician's tibia. Consistent with previous research, our study also measured the highest mean personal dose equivalent values for the tibia of the plate holder during DR of the thoracic limb (2.53 µSv) [18]. Our findings indicate that the technical setup for CT imaging of the horse's distal thoracic limb could be improved to reduce radiation exposure to the gonads. Possible options for reducing radiation exposure to the gonads and therefore minimizing the risk of health consequences include increasing the distance between the CT gantry and the patient by utilizing a longer quick‐release pulley system. The highest amount of scatter radiation is produced between the patient and the radiation source. Following ALARA principles, doubling the distance from the radiation source reduces scatter radiation exposure by a factor of four [38]. Previous studies on performing distal limb CT scans of standing sedated horses demonstrated a reduction in radiation exposure to lead‐equipped personnel, decreasing from 2.7 to 0.5 µSv by increasing the distance from the gantry center from 0.6 to 1.9 m [10]. In the technical setup used in the present study, increasing the distance to the CT gantry would likely reduce radiation exposure to veterinary staff but could also result in less control over the equine patient. Further investigation is needed to determine whether increasing the distance would significantly reduce radiation exposure while still maintaining adequate control over the horse.

Additional precautions to further reduce radiation exposure to veterinary staff include placing a lead screen in front of the operator or lowering the CT radiation dose (mAs) settings [13, 39]. A lead screen has been used to protect personnel during CT scans of the head in standing sedated horses, with exposure values measured at 1 µSv above the thyroid protector and 0 µSv below it. Similarly, a study in human spinal surgery found no measurable radiation exposure to personnel positioned behind a lead screen at a 3 m distance from the cone beam CT [40]. Although this study did not examine the impact of such changes, the authors suggest that the combination of a lead curtain in front of the gantry and a lead screen shielding the veterinary staff would effectively prevent any radiation from passing through. Additionally, if the physical setup allows, the operator could be located outside the CT room, as demonstrated in another study, imaging the front limbs in standing horses by using a long rope to control the horse's limb and, thereby, avoiding any exposure to staff [33].

Overall, the measured exposure doses at various body locations using the MDCT and DR imaging setups for the distal thoracic extremity and tarsus indicated minor radiation levels for the imaging technician when compared with international recommendations. The annual dose limit defined by the International Commission on Radiological Protection would only be reached if a large number of MDCT scans were performed daily on standing horses while the technician remained in the room (i.e., more than 45 scans per day). The maximum number of MDCT scans in standing horses that could be performed per year by a single operator while standing next to the horse before reaching the dose limits was calculated to be 17,857 for the thoracic distal extremity and 29,850 for tarsal MDCTs. For DR imaging, the maximum number of sessions per year for a single operator while standing next to the horse before reaching dose limits was calculated to be 19,607 for the distal thoracic extremity and 22,727 for tarsal examinations (i.e., more than 50 DR sessions per day). Performing this volume of imaging examination each day would be unrealistic. Consequently, the MDCT setup represents a safe diagnostic tool that does not endanger the radiation safety of staff and complies with official dose limits.

In consideration of practicality, the average duration to perform the MDCT scan (MDCT distal thoracic extremity or tarsus) and the DR (DR distal thoracic extremity or tarsus) was recorded in this study. Although the CT scan itself only takes approximately 8 s, the mean duration for completing the scan with the desired image quality was about 20 min. This extended duration was largely due to the need for multiple CT scan repetitions. Repeat scans were necessary because patient movement during image acquisition often resulted in suboptimal image quality. Generally, the frequent repositioning of the horse's limb into the CT gantry consumed most of the time. Based on the authors’ experience, well‐balanced sedation and a moderately tempered horse can shorten the duration of the CT examination. The number of repetitions may also depend on scan duration and the experience of the operators, which may influence their ability to achieve the desired depth of sedation and effectively position the horse. To achieve the desired CT study quality, approximately two repetitions of the entire CT scan were needed. In contrast, for DR, only individual radiographic views needed to be repeated. It is important to note that these repetitions were not included in the dosimetry measurements. Therefore, in a clinical case without any repetitions, the radiation exposure to a single person is as low as indicated by the results of this study. However, considering the average number of scan repetitions required for each imaging tool (two for MDCT and one for DR), the radiation exposure could be three times higher for MDCT or twice as high for DR. Overall, the number of repetitions significantly impacts the cumulative dose for a complete scan, which was not reflected in this study. This means veterinary staff were exposed to clinically relevant higher radiation due to the repetitions. As a result, experienced staff familiar with the technical setup and scanning/positioning procedures are likely exposed to less radiation, closer to the levels observed in this study, compared with trainees or less experienced personnel. The DR image acquisition duration was generally much shorter. This may be due to the greater familiarity of the imaging technician with this procedure and the shorter time required to acquire one image, resulting in less risk of motion‐related artifacts. DR also has other considerable advantages over CT, including the less expensive technical equipment and reduced need for staff training to operate DR. However, the excellent image quality of CT allows for more precise diagnosis [33]. In the author's experience, CT is gradually gaining more popularity among horse owners despite higher expenses, particularly when DR results in inconclusive diagnosis. With recent advancements in CT technology, the overall costs for CT studies in equine patients can be lowered by performing standing examinations instead of general anesthesia [6].

Limitations of this study include the fact that DR and CT repetitions were not included in the dosimetry measurements, which may result in an underestimation of the overall radiation exposure during the complete imaging acquisition. This also means the findings may not accurately reflect exposure levels in typical clinical settings. The radiation exposure values measured during CT image acquisition were obtained using an MDCT scanner equipped with a custom‐designed lead curtain in front of the gantry. Consequently, the results may not fully represent radiation exposure during CT scans conducted without such a lead curtain, limiting the generalizability of the findings. It is likely that radiation exposure to veterinary staff in these cases is higher than reported in our study. Additionally, no iodinated contrast agent was administered during the CT acquisitions, which may further underestimate radiation exposure in clinical situations where contrast administration with additional CT scans is routine.

## 5 Conclusion

In summary, our investigation offers valuable insights into the radiation exposure experienced by veterinary staff during standing equine MDCT and DR procedures and underscores the efficacy of specific interventions in minimizing staff exposure. Our findings demonstrate that implementing a pulley system to increase the distance between the CT scanner and imaging staff and incorporating a curtain to mitigate scatter radiation from the CT scanner can significantly reduce radiation doses to veterinary staff. The data presented herein highlights that the employed MDCT system, coupled with these adaptions, is associated with lower cumulative radiation exposure for veterinary personnel compared with DR procedures performed in the same body area. However, it is noteworthy that radiation readings obtained from the imaging technician's gonad area during the MDCT of the thoracic limb were notably higher than those recorded during DR examinations. This highlights the critical importance of enhanced shielding measures of scatter radiation from the CT scanner, particularly when staff members are present in the room and directly involved in positioning and holding the horse.

## Ethics Statement

This study adheres to international, national, and institutional guidelines for the humane treatment of animals and complies with all relevant legislation. For this study, we ensured the highest standards of veterinary care for the client‐owned horses. Consent from the owners was not required, as the study design did not alter the quality or quantity of diagnostic procedures performed on the patients, thereby posing no harm to the horses. All other relevant details and methodologies are provided in the Materials and Methods section.

## Conflicts of Interest

Tierklinik Luesche GmbH, Germany provided the technical equipment and the patients for the study. The authors declare no conflicts of interest.

## Previous Presentation or Publication Disclosure

No disclosure of previous presentations or publications.

## Reporting Checklist Disclosure

No reporting checklist was used.

## Data Availability

Full sets of data are available from the corresponding author upon request.
